# The Epidemiology and Associated Factors of Ectopic Pregnancy in Saudi Arabia: A Literature Review

**DOI:** 10.1155/ogi/8847352

**Published:** 2026-10-25

**Authors:** Moath Alkathiri, Abdulrahman Almousa

**Affiliations:** ^1^ Department of Family Medicine Ministry of the National Guard Health-Affairs Riyadh Saudi Arabia; ^2^ College of Public Health and Health Informatics King Saud bin Abdulaziz University for Health Sciences Riyadh Saudi Arabia ksau-hs.edu.sa; ^3^ King Abdullah International Medical Research Center (KAIMRC) Riyadh Saudi Arabia kaimrc.med.sa; ^4^ College of Medicine King Saud bin Abdulaziz University for Health Sciences Riyadh Saudi Arabia ksau-hs.edu.sa

**Keywords:** associated factor, ectopic pregnancy, extrauterine pregnancy, prevalence, Saudi Arabia

## Abstract

**Background:**

Ectopic pregnancy (EP) is a serious maternal complication with a worldwide prevalence of about 2%. It is defined as the implantation of the product of conception outside the uterine cavity. Patients with EP may present with a wide range of clinical symptoms, including abdominal/pelvic pain, vaginal bleeding, missed menstrual cycle, and an empty uterine cavity on ultrasound. After diagnosing EPs, medical and/or surgical treatment options are available based on the patient’s hemodynamic status, ectopic location, fetal cardiac activity, size of the gestational sac, level of β‐HCG, and patient profile.

**Objective:**

This narrative literature review aims to summarize published primary studies on the epidemiology and reported associated factors of EP in Saudi Arabia between 2000 and 2024.

**Results:**

A total of 1342 EP cases were reported across the included studies. Reported prevalence estimates ranged from 0.23% to 1.20%. The reviewed studies were from different regions of the Kingdom, including Abha, Khamis Mushait, Yanbu, Makkah, Madina, Jeddah, Khobar, and Majmaah. The prevalence of EPs fluctuated between 2000 and 2019 and had three peaks: 1.13% (2012), 1.14% (2013), and 1.2% (2019). Multiple associated factors linked to EP were reported in this review, including a history of pelvic surgery, C‐sections, previous EP, abortions, intrauterine contraceptive device (IUCD) use, and pelvic inflammatory diseases (PIDs).

**Conclusions:**

EP is a serious complication that causes significant maternal morbidity and mortality and has a substantial burden on the healthcare system. In this review, eleven studies were reviewed regarding the epidemiology of EPs in the Kingdom. No clear pattern or trend in prevalence was identified, although the majority of studies reported lower EP rates than the worldwide prevalence. Several associated factors were reported across the included studies; however, these findings should be interpreted cautiously because most studies were retrospective and descriptive in nature.

## 1. Background

Ectopic pregnancy (EP) is a serious maternal complication with a worldwide prevalence of about 2% [1]. It is defined as the implantation of the product of conception outside the uterine cavity [1]. Abnormal implantation of zygotes can occur at various sites such as ovaries, fallopian tubes, cervix, abdomen, and pelvis [2]. The most common site of extrauterine (ectopic) pregnancy is the fallopian tube [3]. Although clinical advancements and the use of transvaginal ultrasound (TVUS) have led to the early detection of EPs, it is still causing profound maternal morbidity and mortality [3, 4].

Many risk factors have been linked to the development of EPs; however, around 50% of EPs have no identifiable risk factors [1]. Factors such as previous EP, pelvic inflammatory disease (PID), sexually transmitted diseases, fallopian tube damage due to surgery, use of advanced technologies, use of intrauterine devices (IUDs), smoking, and advanced maternal age are all associated with increased risk of EPs [3, 5, 6]. Studies showed that women with a history of previous EPs have a 25% chance of having another extrauterine pregnancy [1]. Also, as maternal age progresses, the risk of EP increases proportionately [5]. The concept behind that is due to the physiological changes in fallopian tubes that somehow impair oocyte transportation and movement [5].

IUDs are considered one the most effective contraceptive methods [1]. However, if women get pregnant while having IUDs, their chances of getting EPs are higher than individuals without IUDs [1]. These observations were found to be higher in hormonal IUDs compared to copper, although the incidence of EPs in individuals with IUDs is only 4% [1].

EP patients may present with abdominal/pelvic pain, vaginal bleeding, missed menstrual cycle, and an empty uterine cavity on ultrasound [4]. These symptoms are usually experienced during the 5^th^ to 10^th^ weeks of gestation, with the pain typically unilateral [4]. The diagnosis of EP can be achieved through careful history taking and physical examination combined with positive laboratory results of beta‐human chorionic gonadotropin **(**β‐HCG) and evidence of an empty uterus by TVUS [2]. TVUS can detect intrauterine pregnancy at a gestational age of 6 weeks and a β‐HCG level of 1500 IU/mL, while abdominal ultrasound can only visualize the product of conception at a higher β‐HCG level (6000–7000 IU/mL) [2]. The absence of intrauterine pregnancy evidenced by ultrasound at the previous β‐HCG levels should raise the suspicion of extrauterine pregnancy [2]. Fortunately, only 15% of EPs were detected after rupture compared to 85% previously [1].

Management of EP depends on the patient’s clinical condition, serum β‐HCG levels, and ultrasound findings. Treatment options include expectant management, medical treatment with methotrexate in selected patients, and surgical intervention when indicated [4]. Because the focus of this review is epidemiology rather than management, a detailed discussion of treatment strategies is beyond the scope of this review. The mortality and morbidity of EPs have been declining due to clinical advancement; however, it is still the leading cause of pregnancy‐related deaths [1]. Although several studies have investigated EP in Saudi Arabia, the findings remain scattered across different regions and healthcare settings, with variations in study design and reported findings. A narrative synthesis of the available evidence may help summarize the epidemiology and reported associated factors of EP in Saudi Arabia, identify knowledge gaps, and inform future research. To our knowledge, no recent narrative review has comprehensively summarized this body of evidence.

## 2. Aim of the Review

The aim of this narrative literature review is to summarize and synthesize published primary studies on the epidemiology and reported associated factors of EP in Saudi Arabia between 2000 and 2024. The inclusion criteria are primary research studies regarding the epidemiology of EPs in Saudi Arabia that were published in English between 2000 and 2024. Secondary research studies, clinical trials, web pages, and book chapters were excluded.

## 3. Methods

This narrative literature review was conducted to summarize published evidence regarding the epidemiology and reported associated factors of EP in Saudi Arabia. The review was designed as a narrative synthesis of the available literature and was not conducted as a systematic review or meta‐analysis. PubMed database was first used to extract relevant articles published between 2000 and 2024. The PubMed search was performed through its electronic search engine using multiple MeSH terms and keywords of three concepts. The first concept is the pathology of interest (keywords: “ectopic pregnancy/Pregnancy, Ectopic” [MeSH]), the second is the country of interest (keywords: “Saudi Arabia/Saudi Arabia” [MeSH]), and the third concept is the epidemiological terms (keywords: “incidence, prevalence, risk, risk factors, prognosis, prognostic factors, trends, course, and patterns”/MeSH terms for each of these words were used). A total of 29 articles were identified by combining the search of the three concepts, and only 7 articles met the inclusion criteria. The inclusion criteria were primary research on the epidemiology of EPs conducted in Saudi Arabia between 2000 and 2024. Secondary researches, clinical trials, web pages, and book chapters were all excluded. To enrich this review and increase the number of articles, another search using Google Scholar was conducted using the term “Ectopic pregnancy” AND “Saudi Arabia.” An additional 4 articles were included through Google Scholar to have a total of 11 articles in this review. The search was conducted between September 24^th,^ 2024, and October 5^th,^ 2024 (Figure 1).

**FIGURE 1:**
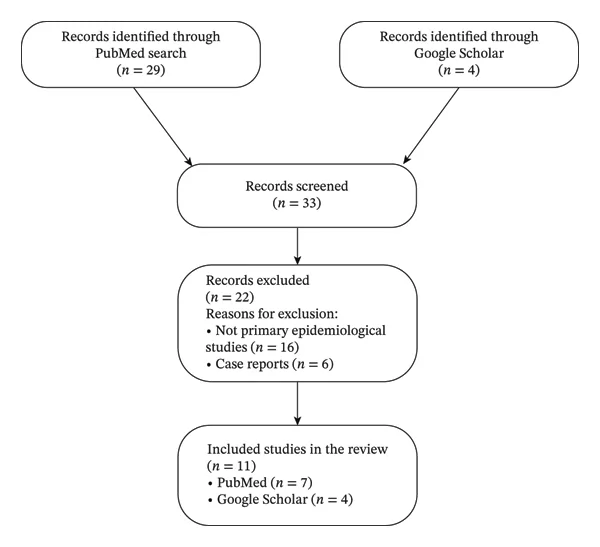
Study selection process. Twenty‐nine studies were identified through PubMed, and four additional studies were identified through Google Scholar. After screening 33 records and excluding 22 studies that did not meet the eligibility criteria, 11 studies were included in the review.

## 4. Quality Assessment

The methodological quality of the included observational studies was assessed using a modified Newcastle–Ottawa Scale (NOS), where sufficient information was available. Studies were evaluated across three domains: selection of participants, comparability or control for confounding, and outcome assessment. Scores ranged from 0 to 9, with scores of 7–9 considered high quality, 4–6 moderate quality, and 0–3 low quality. For one study, only the abstract and article webpage were available; therefore, full quality assessment was not feasible, and this was reported separately. Detailed scoring was performed by the authors based on the modified NOS criteria for observational studies (Table 1).

**TABLE 1:** Methodological quality assessment of included studies using the modified Newcastle–Ottawa Scale (NOS).

Publication year	Author	Design	NOS score	Quality
2000	Archibong and Sobande	Retrospective observational study	5/9	Moderate
2002	Eskandar M et al.	Retrospective observational study	5/9	Moderate
2011	Aziz et al.	Retrospective observational study	5/9	Moderate
2012	Al‐Turki	Retrospective observational study	6/9	Moderate
2013	Saeed J et al	Retrospective descriptive study	5/9	Moderate
2013	Ayaz A et al	Retrospective descriptive study	5/9	Moderate
2013	Al‐Turki	Retrospective trend analysis	6/9	Moderate
2015	Amer et al.	Prospective observational study	6/9	Moderate
2016	Bakhari A et al.	Retrospective observational study	Not fully assessable	Limited full‐text availability
2018	Ellaithy M et al.	Cohort study with follow‐up	8/9	High
2019	Zain H et al.	Retrospective observational study	5/9	Moderate

## 5. Results

Between 2000 and 2019, eleven papers regarding the epidemiology of EPs in the Kingdom were published [6–16]. Although the search covered publications between 2000 and 2024, no eligible studies published after 2019 met the inclusion criteria. A total of 1342 EP cases were reported across the included studies. Reported prevalence estimates ranged from 0.23% to 1.20% (Table 2). The reviewed studies were from different regions of the Kingdom, including Abha, Khamis Mushait, Yanbu, Makkah, Madina, Jeddah, Khobar, and Majmaah. The prevalence of EPs fluctuated between 2000 and 2019 and had three peaks: 1.13% (2012), 1.14% (2013), and 1.2% (2019).

**TABLE 2:** Summaries of data regarding epidemiology of ectopic pregnancy in Saudi Arabia.

Publication year	Author	Region	Number of EPs	Prevalence (%)	Reported associated factors	Location of EP
2000	Archibong and Sobande	Abha	82	0.74	Intrauterine contraceptive devices (IUCDs): 14 (17%) History of ectopic pregnancy: 4 (5%) Infertility treatment: 4 (5%) Pelvic surgery: 3 (4%)	Fallopian tubes: 100%
2002	Eskandar M et al.	Abha	130	0.61	NA	NA
2011	Aziz et al.	Yanbu	66	0.58	Previous abortions: 25 (37.8%) Previous pelvic surgery: 12 (18%) Infertility treatment: 10 (15%) Recurrent ectopic: 6 (9%) IUCD: 3 (4.5%) Fibroid uterus: 2 (3%)	NA
2012	Al‐Turki	Khobar	259	1.13	Previous EP: 31 (12%) C‐Section: 26 (10%) Pregnancy with IVF: 24 (9.3%) Pregnancy with OI: 23 (8.9%) Abortions: 19 (7.3%) IUCD: 11 (4.2%)	NA
2013	Saeed J et al.	Madina	70	0.23	Previous surgical procedures: 32 (45.7%) Miscarriage: 20 (28.5%) Infertility: 8 (11.4%)	Fallopian tubes: 61 (91%) Ovarian: 3 (4.3%) Broad ligament: 2 (2.9%) Abdominal: 1 (1.4%)
2013	Ayaz A et al.	Makkah	44	0.58	Previous pelvic surgery: 13 (29.5%) Infertility treatment: 11 (25%) Pelvic inflammatory disease: 10 (22.7%)	NA
2013	Al‐Turki	Khobar	274	1.14	NA	NA
2015	Amer et al.	Khamis Mushait	25	0.34	NA	NA
2016	Bakhari A et al.	Jeddah	120	NA	History of pelvic surgery: 36 (30%) History of C‐section: 21 (17.5%) Previous ectopic: 10 (8.3%) History of infertility: 9 (7.5%) Current IUCD use: 9 (7.5%)	NA
2018	Ellaithy M et al.	Khamis Mushait	272	NA	Prior miscarriage: 90 (41.5%) Prior laparotomy/CS: 40 (18.4%) Prior infertility: 37 (17%) Prior PID: 12 (5.5%) Induced pregnancy: 6 (2.8%)	Fallopian tubes: 100%
2019	Zain H et al.	Majmaah	NA	1.2	Intrauterine contraceptive devices (23.3%)^∗^ History of PID (9.3%)^∗^ History of ectopic pregnancy (4.7%)^∗^ History of pelvic surgery (2.3%)^∗^	NA

Abbreviations: EP, ectopic pregnancy; IUCD, intrauterine contraceptive device; IVF, in vitro fertilization; NA, not available; PID, pelvic inflammatory disease.

^∗^Exact number of cases was not reported in this study.

Several factors associated with EP were reported in the included studies. Archibong and Sobande reported intrauterine contraceptive device (IUCD) use as the most frequently observed associated factor among women with EP [13]. On the other hand, a study in Yanbu showed that previous abortions and pelvic surgeries were the most predisposing factors for EP [11]. Furthermore, Al‐Turki reported that 31 cases of EP had a previous history, and 26 EP patients had had C‐sections previously [16]. Three studies have agreed that a history of surgical procedures, especially pelvic surgeries, is among the top risk factors [10, 12, 15]. Prior miscarriage, IUCD use, and history of PID were all recorded as risk factors [8, 9].

## 6. Discussion

The worldwide prevalence of EP ranges from 1% to 3%, with a maternal death rate reaching up to 15% [3]. The prevalence of EP in the reviewed articles seems less in the western and southern regions than in the eastern and central areas [6–8, 11–16]. In this review, no pattern or trend can be identified, as the prevalence of EP fluctuated between 0.23% and 1.2% with multiple peaks (Table 2). Compared to the worldwide rate (1%–3%), the majority of studies in this review seem to have a lower rate [6, 7, 9–14].

Other countries reported different trends in EP. In the United States, Hoover et al. showed that the prevalence of EP had not changed during his 6‐year study period [17]. However, another study in the United States demonstrates that the rate of EP has increased from 17.9 per 10,000 to 21.1 per 10,000 [18]. On the contrary, EP rates in Norway have declined from 16 per 10,000 to 8.4 per 10,000 between 1970 and 2004 [19]. This decline in EP was also noticed in the United Kingdom [20]. This decline may be attributed to increased use of hormonal contraceptives and reduction of sexually transmitted diseases, thus reducing PID [19].

Several factors have been reported to be associated with EP in previous studies; however, these factors are only present in 25%–50% of cases [13]. Identifying such factors may help in early detection and, thus, intervention, and that would reduce maternal morbidity and mortality [12]. Data regarding the association between the use of IUCDs are different among studies [21]. Archibong and Sobande showed that IUCD use accounted for 17% of EP cases [13]. A higher percentage (23.3%) was reported in a study conducted in Al Majmaah [8]. In France, the use of IUDs is associated with one‐quarter of all EP cases [21]. In general, the use of IUCDs lowers the chance of fertilization and, hence, pregnancy [21]. However, if women with IUCDs get pregnant, the chance of developing extrauterine pregnancies is higher [21].

The reviewed studies reported several factors that were frequently observed among women with EP. Previous surgical procedures were among the most frequently reported associated factors across several included studies [7, 10, 12]. This association might be explained by the damage a pelvic surgery does to the fallopian tube, thus disrupting the anatomy [4]. Also, a similar explanation is applied to the relationship between PID and EPs [4]. PID was reported among the top risk factors in two studies in this review [8, 12]. Other commonly reported associated factors included a previous history of EP and abortion, which were observed in seven studies included in this review [7–11, 13, 16].

Advanced maternal age has been reported in the literature as a potential risk factor for EP, although findings have not been consistent across studies [11]. A study conducted in Norway stated that the overall population rate of extrauterine pregnancy was 1.8%; however, for females older than 35 years, the reported rate was 4.1% [22]. Similar observations were reported in some of the studies included in this review [7, 11]. Regarding the location of EP, fallopian tubes were the most commonly reported area of extrauterine pregnancies in this review [7, 9, 13].

### 6.1. Limitations

This review has a few limitations that can be stated. One of which is the possibility of missing papers that were published in nonindexed journals. Also, most included studies were retrospective and descriptive in nature, which increases the risk of selection bias, information bias, and residual confounding. Therefore, the reported associated factors should be interpreted cautiously, as most studies did not provide adjusted estimates or formal risk analyses. Moreover, the small sample size of some studies may affect the accuracy of their conclusion.

## 7. Conclusion

In conclusion, EP is a serious complication that causes significant maternal morbidity and mortality and has a substantial burden on the healthcare system. In this review, eleven studies were reviewed regarding the epidemiology of EPs in the Kingdom. No pattern or trend in the prevalence can be detected, although the majority of studies in this review reported a lower rate of EP compared to the worldwide prevalence rate. Several associated factors were reported across the included studies, particularly prior pelvic surgery, previous EP, abortion history, IUCD use, and PID. Large prospective multicenter studies should be conducted in Saudi Arabia to study this topic in more detail and provide further recommendations to minimize maternal morbidity and mortality.

**Nomenclature**
EP: Ectopic pregnancy
IUCD: Intrauterine contraceptive device
IVF: In vitro fertilization
OI: Ovulation induction
PID: Pelvic inflammatory disease
TVUS: Transvaginal ultrasound
β‐HCG: Beta‐human chorionic gonadotropin
NA: Not available

## Funding

No funding was required.

## Conflicts of Interest

The authors declare no conflicts of interest.

## Data Availability

Data sharing is not applicable to this article as no new data were created or analyzed in this study.
